# (Furan-2-yl)[(furan-2-yl)carbonyl­disul­fanyl]methanone

**DOI:** 10.1107/S160053681104356X

**Published:** 2011-10-29

**Authors:** Qian Wang, Youqin Shu, Xuehui Hou

**Affiliations:** a Radio and TV University of Henan, Zhengzhou 450008, People’s Republic of China; b Department of Quality Detection and Management, Zhengzhou College of Animal Husbandry Engineering, Zhengzhou 450011, People’s Republic of China

## Abstract

The mol­ecule of the title compound, C_10_H_6_O_4_S_2_, has crystallographically imposed twofold symmetry. The dihedral angle formed by the furan rings is 80.90 (8)°. In the crystal, mol­ecules are linked by weak C—H⋯O hydrogen bonds into chains running parallel to the *a* axis [C—S—S—C torsion angle = 82.04 (11)°].

## Related literature

For the applications of furan-2-carbothioic-S-acid, see: Deshpande *et al.* (2004 ▶); Stoll *et al.* (1967 ▶).
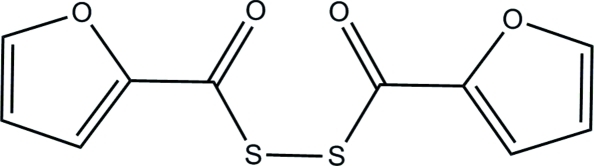


## Experimental

### 

#### Crystal data

C_10_H_6_O_4_S_2_
*M* *_r_* = 254.29
Orthorhombic, 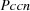

*a* = 13.6900 (13) Å
*b* = 7.9611 (7) Å
*c* = 9.9042 (10) Å
*V* = 1079.43 (18) Å^3^
*Z* = 4
Mo *K*α radiation
μ = 0.49 mm^−1^
*T* = 298 K
0.41 × 0.39 × 0.30 mm

#### Data collection

Bruker SMART CCD area-detector diffractometer
Absorption correction: multi-scan (*SADABS*; Sheldrick, 1996 ▶) *T*_min_ = 0.826, *T*_max_ = 0.868
3627 measured reflections
952 independent reflections
750 reflections with *I* > 2σ(*I*)
*R*_int_ = 0.028

#### Refinement

*R*[*F* ^2^ > 2σ(*F* ^2^)] = 0.032
*wR*(*F* ^2^) = 0.103
*S* = 1.00
952 reflections
74 parameters
H-atom parameters constrained
Δρ_max_ = 0.17 e Å^−3^
Δρ_min_ = −0.23 e Å^−3^

### 

Data collection: *SMART* (Siemens, 1996 ▶); cell refinement: *SAINT* (Siemens, 1996 ▶); data reduction: *SAINT*; program(s) used to solve structure: *SHELXS97* (Sheldrick, 2008 ▶); program(s) used to refine structure: *SHELXL97* (Sheldrick, 2008 ▶); molecular graphics: *SHELXTL* (Sheldrick, 2008 ▶); software used to prepare material for publication: *SHELXTL*.

## Supplementary Material

Crystal structure: contains datablock(s) I, global. DOI: 10.1107/S160053681104356X/rz2653sup1.cif

Structure factors: contains datablock(s) I. DOI: 10.1107/S160053681104356X/rz2653Isup2.hkl

Supplementary material file. DOI: 10.1107/S160053681104356X/rz2653Isup3.cml

Additional supplementary materials:  crystallographic information; 3D view; checkCIF report

## Figures and Tables

**Table 1:** Hydrogen-bond geometry (Å, °)

*D*—H⋯*A*	*D*—H	H⋯*A*	*D*⋯*A*	*D*—H⋯*A*
C4—H4⋯O2^i^	0.93	2.57	3.463 (3)	162

Symmetry code: (i) 
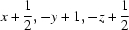
.

**Fig. 1.**
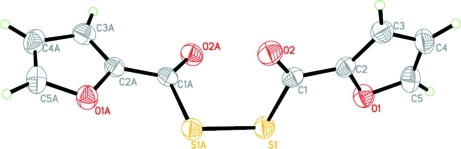
The molecular structure of the title compound, with 50% probability displacement ellipsoids.

**Fig. 2.**
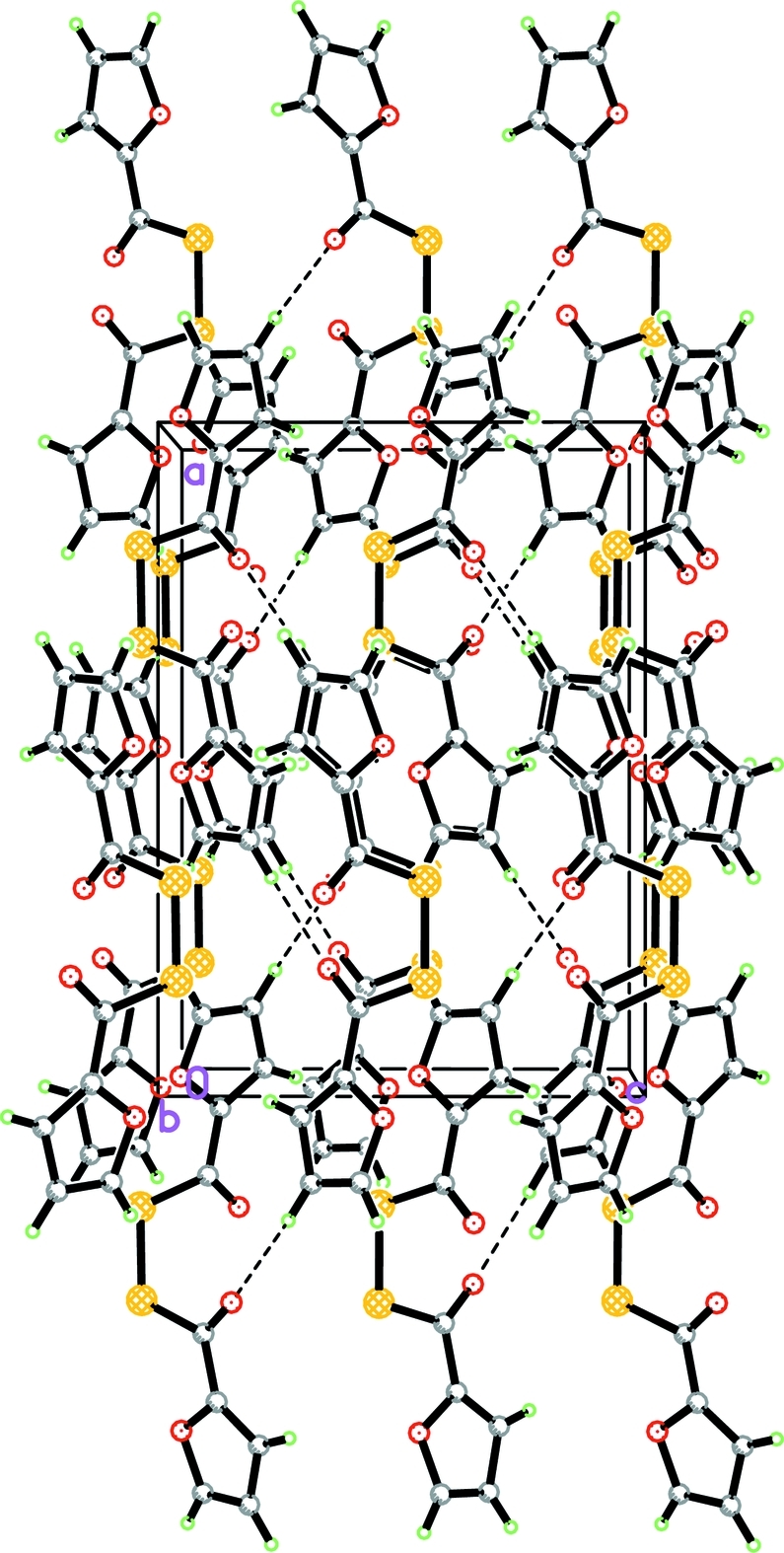
Crystal packing of the title compound viewed along the a axis. INtermolecular hydrogen bonds are shown as dashed lines.

## supplementary crystallographic information

### Comment

The title compound is a dimeric form of furan-2-carbothioic-S-acid, which has a broad spectrum of applications in the fields of medicinal chemistry (Deshpande *et al.*, 2004) and food additives (Stoll *et al.*, 1967). As a contribution in this field, we report here the crystal structure of the title compound.

The molecular structure of the title compound is shown in Fig. 1. The molecule has crystallographically imposed twofold axis. The furan rings are oriented to form a dihedral angle of 80.90 (8)°. In the crystal structure (Fig. 2), molecules are linked by weak intermolecular C—H···O hydrogen bonds (Table 1) forming chains parallel to the *a* axis.

### Experimental

To a solution of furan-2-carboxylic acid (11.2 g, 0.10 mol) in dioxane, NaHS (11.2 g, 0.20 mol) was added. The mixture was stirred at 50°C for 4 h. Then mixture was concentrated and purified by crystallization from ethyl acetate. Colourless crystals suitable for X-ray analysis were obtained on slow evaporation of the solvent.

### Refinement

All H atoms were placed geometrically and treated as riding on their parent atoms, with C—H = 0.93–0.96 Å and *U*_iso_(H) = 1.2*U*_eq_(C) or 1.5*U*_eq_(C) for methyl H atoms.

### Crystal data

**Table:**

C_10_H_6_O_4_S_2_	*F*(000) = 520
*M**_r_* = 254.29	*D*_x_ = 1.565 Mg m^−^^3^
Orthorhombic, *P**c**c**n*	Mo *K*α radiation, λ = 0.71073 Å
Hall symbol: -P 2ab 2ac	Cell parameters from 1606 reflections
*a* = 13.6900 (13) Å	θ = 2.6–25.7°
*b* = 7.9611 (7) Å	µ = 0.49 mm^−^^1^
*c* = 9.9042 (10) Å	*T* = 298 K
*V* = 1079.43 (18) Å^3^	Block, colourless
*Z* = 4	0.41 × 0.39 × 0.30 mm

### Data collection

**Table:**

Bruker SMART CCD area-detector diffractometer	952 independent reflections
Radiation source: fine-focus sealed tube	750 reflections with *I* > 2σ(*I*)
graphite	*R*_int_ = 0.028
φ and ω scans	θ_max_ = 25.0°, θ_min_ = 3.0°
Absorption correction: multi-scan (*SADABS*; Sheldrick, 1996)	*h* = −16→9
*T*_min_ = 0.826, *T*_max_ = 0.868	*k* = −7→9
3627 measured reflections	*l* = −11→11

### Refinement

**Table:**

Refinement on *F*^2^	Secondary atom site location: difference Fourier map
Least-squares matrix: full	Hydrogen site location: inferred from neighbouring sites
*R*[*F*^2^ > 2σ(*F*^2^)] = 0.032	H-atom parameters constrained
*wR*(*F*^2^) = 0.103	*w* = 1/[σ^2^(*F*_o_^2^) + (0.0569*P*)^2^ + 0.4883*P*] where *P* = (*F*_o_^2^ + 2*F*_c_^2^)/3
*S* = 1.00	(Δ/σ)_max_ < 0.001
952 reflections	Δρ_max_ = 0.17 e Å^−^^3^
74 parameters	Δρ_min_ = −0.23 e Å^−^^3^
0 restraints	Extinction correction: *SHELXL97* (Sheldrick, 2008), Fc^*^=kFc[1+0.001xFc^2^λ^3^/sin(2θ)]^-1/4^
Primary atom site location: structure-invariant direct methods	Extinction coefficient: 0.026 (3)

### Special details

**Table:**

Geometry. All e.s.d.'s (except the e.s.d. in the dihedral angle between two l.s. planes) are estimated using the full covariance matrix. The cell e.s.d.'s are taken into account individually in the estimation of e.s.d.'s in distances, angles and torsion angles; correlations between e.s.d.'s in cell parameters are only used when they are defined by crystal symmetry. An approximate (isotropic) treatment of cell e.s.d.'s is used for estimating e.s.d.'s involving l.s. planes.
Refinement. Refinement of *F*^2^ against ALL reflections. The weighted *R*-factor *wR* and goodness of fit *S* are based on *F*^2^, conventional *R*-factors *R* are based on *F*, with *F* set to zero for negative *F*^2^. The threshold expression of *F*^2^ > σ(*F*^2^) is used only for calculating *R*-factors(gt) *etc*. and is not relevant to the choice of reflections for refinement. *R*-factors based on *F*^2^ are statistically about twice as large as those based on *F*, and *R*- factors based on ALL data will be even larger.

### Fractional atomic coordinates and isotropic or equivalent isotropic displacement parameters (Å^2^)

**Table:**

	*x*	*y*	*z*	*U*_iso_*/*U*_eq_	
S1	0.32229 (4)	0.22302 (9)	0.54908 (7)	0.0562 (3)	
O1	0.51977 (13)	0.2344 (2)	0.46510 (19)	0.0596 (5)	
O2	0.30932 (12)	0.4503 (2)	0.35136 (19)	0.0651 (6)	
C1	0.36270 (17)	0.3584 (3)	0.4132 (2)	0.0483 (6)	
C3	0.5256 (2)	0.4169 (3)	0.2957 (3)	0.0621 (7)	
H3	0.5075	0.4955	0.2308	0.074*	
C2	0.46678 (16)	0.3429 (3)	0.3863 (2)	0.0478 (6)	
C4	0.6204 (2)	0.3519 (4)	0.3184 (3)	0.0683 (8)	
H4	0.6769	0.3799	0.2713	0.082*	
C5	0.6134 (2)	0.2437 (4)	0.4195 (3)	0.0676 (8)	
H5	0.6654	0.1823	0.4545	0.081*	

### Atomic displacement parameters (Å^2^)

**Table:**

	*U*^11^	*U*^22^	*U*^33^	*U*^12^	*U*^13^	*U*^23^
S1	0.0521 (4)	0.0597 (5)	0.0569 (5)	−0.0007 (3)	−0.0038 (3)	0.0117 (3)
O1	0.0531 (10)	0.0581 (11)	0.0676 (12)	0.0036 (8)	−0.0012 (8)	0.0087 (8)
O2	0.0632 (11)	0.0682 (12)	0.0640 (11)	0.0093 (9)	−0.0095 (9)	0.0170 (9)
C1	0.0548 (13)	0.0449 (13)	0.0452 (12)	−0.0018 (11)	−0.0073 (11)	−0.0034 (10)
C3	0.0696 (17)	0.0600 (15)	0.0566 (15)	−0.0131 (13)	−0.0038 (13)	0.0064 (12)
C2	0.0516 (13)	0.0438 (13)	0.0479 (13)	−0.0037 (11)	−0.0064 (11)	−0.0034 (10)
C4	0.0555 (16)	0.0747 (19)	0.0747 (19)	−0.0175 (14)	0.0093 (14)	−0.0103 (16)
C5	0.0477 (14)	0.0661 (17)	0.089 (2)	0.0021 (12)	−0.0003 (15)	−0.0066 (16)

### Geometric parameters (Å, °)

**Table:**

S1—C1	1.811 (2)	C3—C2	1.341 (3)
S1—S1^i^	2.0254 (12)	C3—C4	1.416 (4)
O1—C5	1.361 (3)	C3—H3	0.9300
O1—C2	1.372 (3)	C4—C5	1.324 (4)
O2—C1	1.202 (3)	C4—H4	0.9300
C1—C2	1.455 (3)	C5—H5	0.9300
			
C1—S1—S1^i^	99.92 (8)	C3—C2—C1	132.3 (2)
C5—O1—C2	106.0 (2)	O1—C2—C1	117.8 (2)
O2—C1—C2	123.6 (2)	C5—C4—C3	106.9 (3)
O2—C1—S1	123.74 (19)	C5—C4—H4	126.5
C2—C1—S1	112.64 (17)	C3—C4—H4	126.5
C2—C3—C4	106.5 (2)	C4—C5—O1	110.8 (3)
C2—C3—H3	126.8	C4—C5—H5	124.6
C4—C3—H3	126.8	O1—C5—H5	124.6
C3—C2—O1	109.9 (2)		
			
S1^i^—S1—C1—O2	1.2 (2)	S1—C1—C2—C3	179.3 (2)
S1^i^—S1—C1—C2	−178.38 (15)	O2—C1—C2—O1	179.5 (2)
C4—C3—C2—O1	0.0 (3)	S1—C1—C2—O1	−1.0 (3)
C4—C3—C2—C1	179.7 (2)	C2—C3—C4—C5	0.2 (3)
C5—O1—C2—C3	−0.2 (3)	C3—C4—C5—O1	−0.4 (3)
C5—O1—C2—C1	180.0 (2)	C2—O1—C5—C4	0.4 (3)
O2—C1—C2—C3	−0.3 (4)		

Symmetry codes: (i) −*x*+1/2, −*y*+1/2, *z*.

### Hydrogen-bond geometry (Å, °)

**Table:**

*D*—H···*A*	*D*—H	H···*A*	*D*···*A*	*D*—H···*A*
C4—H4···O2^ii^	0.93	2.57	3.463 (3)	162.

Symmetry codes: (ii) *x*+1/2, −*y*+1, −*z*+1/2.
